# Dry Season Determinants of Malaria Disease and Net Use in Benin, West Africa

**DOI:** 10.1371/journal.pone.0030558

**Published:** 2012-01-23

**Authors:** Nicolas Moiroux, Olayidé Boussari, Armel Djènontin, Georgia Damien, Gilles Cottrell, Marie-Claire Henry, Hélène Guis, Vincent Corbel

**Affiliations:** 1 MIVEGEC (IRD 224-CNRS 5290-UM1), Institut de Recherche pour le Développement (IRD), Cotonou, Bénin; 2 Centre de Recherche en Entomologie de Cotonou (CREC), Ministère de la Santé, Cotonou, Bénin; 3 UMR216 Mère et Enfant Face aux Infections Tropicales, Institut de Recherche pour le Développement (IRD), Cotonou, Bénin; 4 Institut des Sciences Biomédicales Appliquées (ISBA), Université d'Abomey Calavi, Cotonou, Bénin; 5 Service de Coopération et d'Action Culturelle (SCAC), Ambassade de France, Cotonou, Bénin; 6 UMR CMAEE, CIRAD, Montpellier, France; Instituto de Higiene e Medicina Tropical, Portugal

## Abstract

**Background:**

To achieve malaria eradication, control efforts have to be sustained even when the incidence of malaria cases becomes low during the dry season. In this work, malaria incidence and its determinants including bed net use were investigated in children of under 5 years of age in 28 villages in southern Benin during the dry season.

**Methods and Findings:**

Mean malaria clinical incidence was measured in children aged 0–5 years by active case detection in 28 villages of the Ouidah-Kpomasse-Tori Bossito sanitary district between November 2007 and March 2008. Using Poisson mixed-effect models, malaria incidence was assessed according to the level of transmission by different vector species, and Long-Lasting Insecticide-treated mosquito Nets (LLIN) use and ownership. Then, a Binomial mixed-effect model was developed to assess whether nighttime temperature (derived from MODIS remote sensing data), biting nuisance and LLIN ownership are good predictors of LLIN use >60%. Results suggested that *Anopheles funestus* (Incidence Rates Ratio (IRR) = 3.38 [IC95 1.91–6]) rather than *An. gambiae s.s.* is responsible for malaria transmission. A rate of LLIN use >60% was associated with a lower risk of malaria (IRR = 0.6 [IC95 0.37–0.99]). Low nocturnal temperature and high biting nuisance were good predictors of LLIN use >60%.

**Conclusions:**

As recommended by the Malaria Eradication (MalERA) Consultative Group on Modelling, there is a need to understand better the effects of seasonality on malaria morbidity. This study highlights the need to take into account the specificity of malaria epidemiology during the dry-hot season and get a better understanding of the factors that influence malaria incidence and net use. These findings should help National Malaria Control Programmes to implement more effective and sustainable malaria control strategies in Africa.

## Introduction

Increased international funding of malaria control has resulted in a global decrease of the number of cases and deaths since 2000 [1]. In particular, the scaling up of Long-Lasting Insecticide-treated mosquito Nets (LLIN) and Indoor Residual Spraying (IRS) programmes has contributed to the idea that malaria eradication may be feasible [2]. Unfortunately, recent increases in malaria cases were recorded in some African countries indicating the tenuousness of malaria control in this part of the world [1], [3].

Moreover, seasonal climatic change is known to be an important determinant of malaria incidence [4] since climate conditions both mosquito vector dynamics and parasite development rates [5]. Malaria incidence is usually low during the dry-hot season when vector populations are reduced and spatially confined. In consequence, most studies focus on the peak transmission season (i.e., the rainy season) and the epidemiological picture during the dry-hot season is often neglected. However, if malaria is to be eradicated, it will be essential to sustain vector control efforts through the dry-hot season when vector density is low. Moreover, the dry-hot season is important because the vector profile may change and influence the pattern of transmission. In Benin, comparison of entomological data from two studies [6], [7] carried out in the mesoendemic Ouidah-Kpomassè-Tori Bossito (OKT) region suggests that *An. funestus* partially replaces the main malaria vector *An. gambiae s.s.* during the dry-hot season. The switch from *An. gambiae s.s.* to other anopheline species such as *An. funestus*, *An. moucheti* and *An. melas* has been previously documented in Africa [8], [9] and this is particularly worrying because our understanding of the behaviour, ecology and insecticide resistance of these species is limited [3], [10].

Furthermore, some studies have shown that mosquito nets are less likely to be used during the dry-hot season [6], [11], [12] because of the discomfort of having to sleep under a net on a hot night [12], [13] and when the mosquito biting nuisance is low [12], [14], [15]. In Southern Benin, a longitudinal survey conducted in 7 villages of the OKT district reported an average decrease of 31% in net use during the dry season even though most families (over 80%) had nets available throughout the study [6]. Major disparities were observed in both the density and diversity of culicidae [7] and this could have influenced LLIN use and the risk of malaria attacks in people from different villages.

In this context, we investigated the factors that determine the likelihood of malaria attacks in children of under 5 years of age in the OKT district during the dry season. A poisson mixed-effect model was used to assess relationships between malaria incidence and transmission by different vector species, LLIN use and net ownership. A binomial mixed-effect model was then developed to asses whether night-time temperature (derived from MODIS remote sensing data), biting nuisance and LLIN ownership predict LLIN use >60%. Better understanding of the factors that determine malaria incidence and net use during the dry-hot season would help National Malaria Control Programs (NMCPs) to implement more effective and sustainable malaria control strategies in Africa.

## Methods

### Ethics Statement

The IRD (Institut de Recherche pour le Développement) Ethics Committee and the National Research Ethics Committee of Benin approved the study (CNPERS, reference number IRB00006860). All necessary permits were obtained for the described field studies. No mosquito collection was done without the approval of the village chief, the owner and occupants of the collection house. Mosquito collectors gave their written informed consent and were treated free of charge for malaria presumed illness throughout the study. They were also vaccinated against yellow fever. Before the study, the head of the family (or the guardian) of the selected child gave their written informed consent. During the monitoring periods, all children of villages, whether participating or not in the study, were treated free of charge by the medical staff.

### Study area

This study was carried out in the OKT health administrative region in southern Benin (on the Atlantic coast) following the nationwide distribution of LLINs to children of under 5 years of age by the NMCP [6], [7]. The local climate is coastal-guinean with four seasons including a long dry season (between November and March) which is the hottest of the year (with average monthly temperatures close to 28°C). Average annual rainfall was 1,100 mm in 2007–2008 with less than 10% falling during the long dry season. Of the 58 villages screened at the baseline, 28 were enrolled. 30 were excluded because they did not fulfil inclusion criteria i.e. distance between two villages of more than 2 km, population size between 250 and 500 inhabitants with non-isolated habitations and absence of any local health care centre. Investigations were conducted in 28 villages selected on the basis of a population size between 250 and 500 inhabitants, a distance between two villages of more than 2 km and the absence of any local health care center [6], [7].

### Entomological data

Mosquitoes were collected by human landing catches for three surveys conducted every six weeks between November 2007 and March 2008. Mosquitoes were collected from 10 p.m. to 6 a.m. both indoors and outdoors at four sites in each village on two successive nights for each survey (i.e., 16 person-nights per village per survey). Malaria vectors belonging to the *Anopheles gambiae* complex and the *Anopheles funestus* group were identified to species by PCR and processed for circumsporozoite protein (CSP) ELISA detection of *Plasmodium falciparum* sporozoites [7]. Human Biting Rates (HBR) and Entomological Inoculation Rates (EIR) were calculated for each vector species and HBR was also calculated for all culicidae to estimate the overall mosquito-biting nuisance in this area.

### Clinical malaria, LLIN use and LLIN ownership data

In each of the 28 villages, a cohort of 60 children of between zero and five years of age was randomly selected (on the based of a comprehensive census carried out in 2007 to identify the number of houses, beds and sleeping mats). Active Case Detection (ACD) for malaria attacks was carried out over three six-day periods six weeks apart, 14 days after mosquito collection.

A malaria attack was defined by a high axillary temperature (≥37.5°C), fever in the 48 hours preceding the first day of ACD, or any sign suggestive of malaria associated with a parasitaemia of over 1,999 asexual forms of *P. falciparum* per microlitre of blood [6].

The incidence of clinical malaria was calculated by dividing the number of pathological episodes attributed to malaria by the number of child-days surveyed. The ownership and use of LLINs (Permanet® 2.0) which had been distributed in October 2007 by the NMCP, were evaluated among the children of the cohort at the same time as ACD. Unannounced visits were done by a nurse around 9 p.m. (when children were expected to be asleep) to control whether the LLIN is present (ownership) and whether children are sleeping under it (use). Rates of LLIN ownership and use were both calculated with respect to the total number of observations. Details on the methods used for sampling, data collection and clinical measurements have been published [6].

### Temperature data

We used the Land-Surface Temperature (LST) at a spatial resolution of one kilometer measured by the Moderate Resolution Imaging Spectroradiometer (MODIS) sensors on the Terra satellite (https://lpdaac.usgs.gov/). The average 8-day nocturnal temperature at the coordinates of each village (georeferenced using the Global Positioning System) was extracted using ArcGis ArcInfo 9.3 software (ESRI, Redlands, CA). The data were then converted into Celsius. Nocturnal LST data are known to provide good estimates of minimum temperatures in Africa in all ecosystems [16].

### Incidence of clinical malaria

The dependent variable was mean clinical malaria incidence and the explanatory variables were the EIR of each vector species (categorized in two classes: null and positive), LLIN use and LLIN ownership (both categorized in two classes using respective thresholds of 60% and 80%). The threshold of 80% for LLIN ownership was adopted because it is considered by the World Health Organization (WHO) as the minimum objective for effective community protection against malaria [1]. For LLIN use, the threshold of 60% was selected because this level was approved in the Abuja declaration as a feasible objective to prevent malaria infection and suffering [17]. Relationships between the dependent variable and each explanatory variable were assessed using an univariate Poisson Mixed Effect (PME) model with a random intercept (taking into account possible correlations associated with repeated measurements in each village). We then investigated the combined effects of the explanatory variables on the occurrence of malaria attacks using a multivariate PME.

### LLIN use

Relationships between the dependant variable, LLIN use rate (categorized in two classes), and biting nuisance, nocturnal temperature (both categorized in four quartiles), and LLIN ownership were assessed by univariate analysis using a Binomial Mixed Effect (BME) model with a random intercept. A multivariate BME model was then developed to assess the combined effects of the three covariates on the probability of a rate of LLIN use of over 60%.

Statistical analyses were conducted using R-software [18] with additional functions from the ‘lme4’ [19] package.

## Results

### Incidence of clinical malaria and transmission

**Figure 1:**
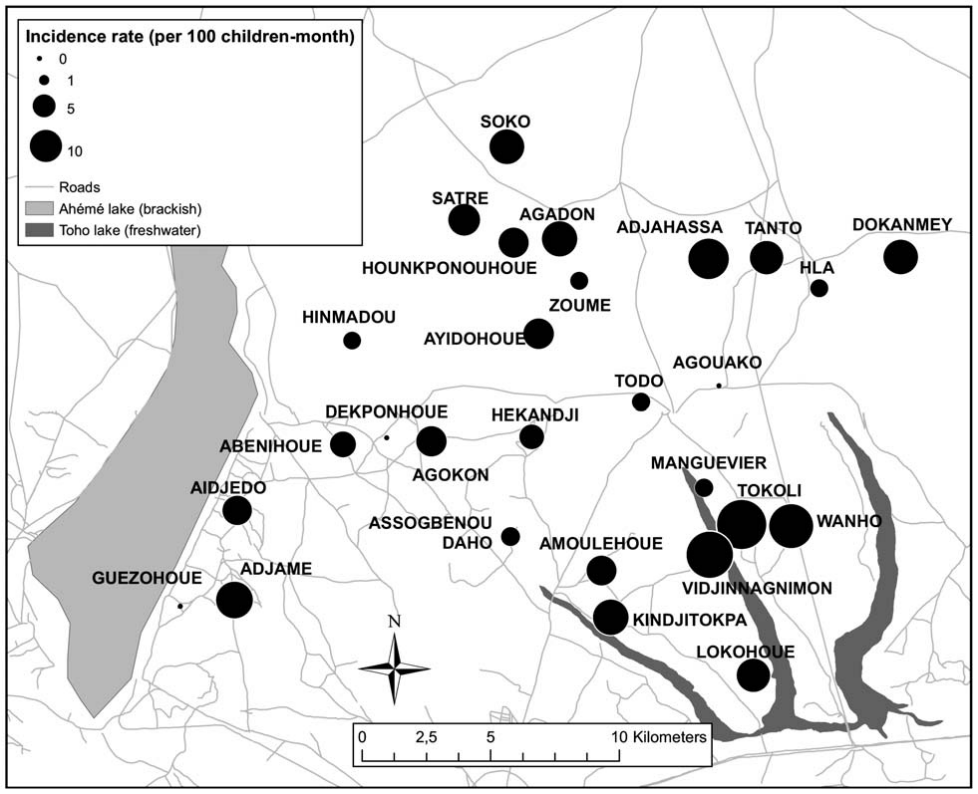
Map showing malaria incidence in children of under 5 years of age in 28 villages in Southern Benin during the dry season.

During the three surveys, 27,265 children-day were followed up and a total of 79 malaria attacks were recorded. This represents an average of 8.8 (95% CI 6.7–10.9) malaria cases per 100 child-months. Figure 1 shows the distribution of clinical malaria in the 28 villages. Great disparities were observed between different villages with no cases at all detected in Agouako, Guezohoue or Dekponhoue compared with 23 cases per 100 children-month in Tokoli alone. 13,822 culicidae mosquitoes were caught during 1,344 human-nights corresponding to an average HBR of 1,028 (95% CI 736–1320) bites per human per 100 nights. Two malaria vector species were found coexisting, namely *Anopheles gambiae s.s.* (n = 94) and *Anopheles funestus* (n = 381). The average HBR was 7.0 (95% CI 3.4–10.6) per human per 100 nights for *An. gambiae s.s.*, and 28.4 (95% CI 10.6–42.6) for *An. funestus*. Only ten *An. funestus* individuals and five *An. gambiae s.s.* were found to be positive for CSP, corresponding to an average EIR of 1.12 (95% CI 0.29–1.95) infected bites per human per 100 nights. Mean percentages of LLIN ownership and use were 77.7% (95% CI 75.2–80.2) and 51.9% (95% CI 46.9–55.9) respectively. The average nocturnal temperature was 20.6°C (95% CI 20.2–21).

### Factors influencing the incidence of clinical malaria during the dry season

**Figure 2:**
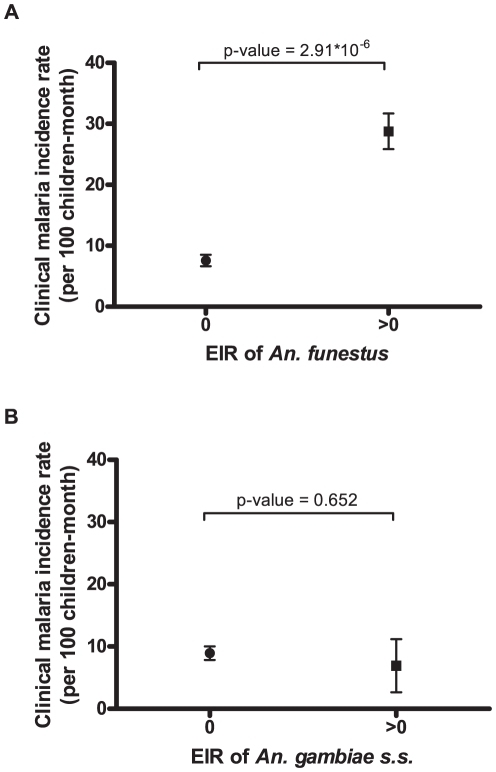
Mean village-survey malaria incidence in children of under 5 years of age for zero and positive EIRs of (a) *An. funestus* and (b) *An. gambiae s.s.* Error bars represent standard error. EIR: Entomological Inoculation Rate. P-value given according to Poisson Mixed-Effect models taking into account repeated measurements.

**Figure 3:**
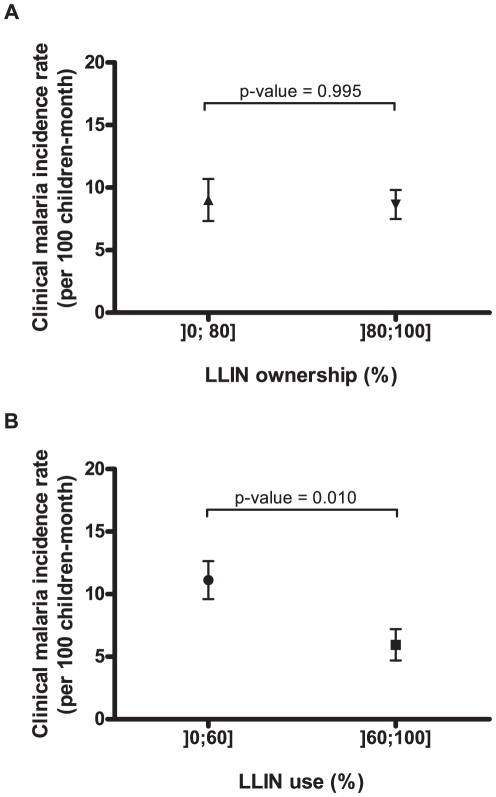
Mean village-survey malaria incidence in children of under 5 years of age for (a) >80% LLIN ownership and (b) >60% LLIN use. Error bars represent standard error. LLIN: Long Lasting Insecticidal Net. P-value given according to Poisson Mixed-Effect models taking into account repeated measurements.

Malaria incidence (MI) correlated closely (p<0.0001, according to PME) with positive EIR of *An. funestus* but not *An. gambiae s.s.* (Figure 2a, 2b). Moreover, univariate analysis showed that MI was not significantly reduced for >80% LLIN ownership (p = 0.995; figure 3a) whereas it was significantly lower for >60% LLIN use (p = 0.010; Figure 3b).

Multivariate PME analysis first confirmed that the component of transmission due to *An. funestus* significantly correlated with malaria incidence (Table 1) and that a rate of LLIN use of over 60% provided significant protection against malaria attacks in children of under 5 years of age.

**Table 1:** Poisson Mixed-Effects model to study malaria incidence in children of under 5 years of age (N = 84; number of groups = 28), according to vector transmission and LLIN use rate.

	Univariate analysis				Multivariate analysis			
Effects	Incidence Rates Ratio	95% Confidence interval		P-value	Incidence Rates Ratio	95% Confidence interval		P-value
EIR of *An. funestus* ‡ $								
0 (79)	1				1			
>0 (5)	**3.83**	2.18	6.71	**2.91e-6**	**3.382**	1.91	6.00	**0.00003**
EIR of *An. gambiae* ‡ $								
0 (80)	1				1			
>0 (4)	0.77	0.24	2.43	0.652	1.067	0.33	3.45	0.913
LLIN use § $								
≤60% (47)	1				1			
>60% (37)	**0.53**	0.33	0.86	**0.010**	**0.602**	0.37	0.99	**0.044**

‡ EIR: Entomological Inoculation Rate.

§ LLIN: Long Lasting Insecticidal Net.

$ the size of each category is indicated in parentheses.

### Factors influencing LLIN use

**Figure 4:**
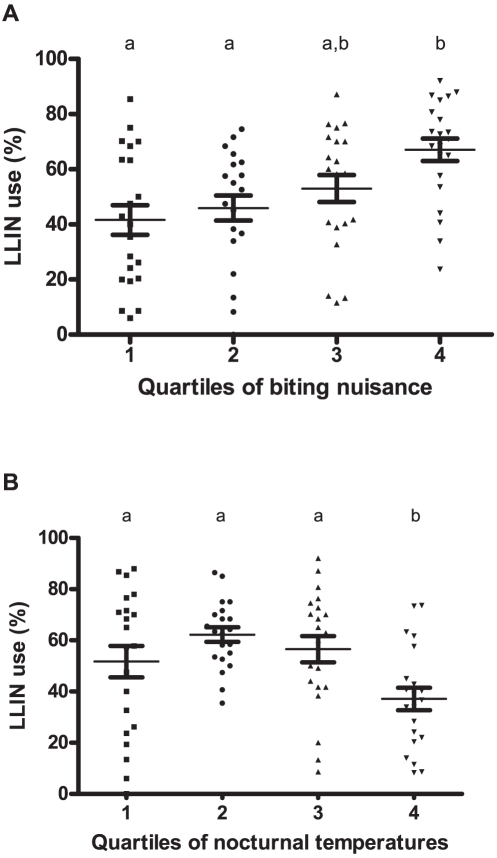
Mean village-survey LLIN use rates in children of under 5 years of age according to the quartiles of (a) biting nuisance and (b) nocturnal temperature. Quartiles of biting nuisance are 1: ]6;56], 2: ]56;447], 3: ]447;1456], 4: ]1456;6419] in bites of mosquitoes per human per 100 nights. Quartiles of temperature are 1: ]14.1;19.5], 2: ]19.5;20.5], 3: ]20.5;22], 4: ]22;23.7] in Celsius degrees. Error bars represent standard error. Quartiles carrying the same letter are not significantly different (p-value<0.05) analysing the probability of LLIN use >60% (binomial mixed-effect model taking into account repeated measurements). LLIN: Long Lasting Insecticidal Net.

At high levels of nuisance (quartile 4; HBR >1,456 bites per human per 100 nights), the probability of LLIN use >60% was significantly higher (BME p-values<0.05) than in the two lower quartiles (Figure 4a). On the other hand, no correlation (BME p-value = 0.281) was found between the probability of LLIN use >60% and the HBR for all vectors (i.e. the part of the nuisance due to malaria vectors).

Probability of LLIN use >60% was negatively associated with nocturnal temperature (BME p-values<0.05) showing that, as the temperature increases, LLIN use decreases. The figure 4b shows that LLIN use decreased to 37.1% [95% CI 27.9–46.3] for temperatures above 22°C (the fourth quartile).

**Figure 5:**
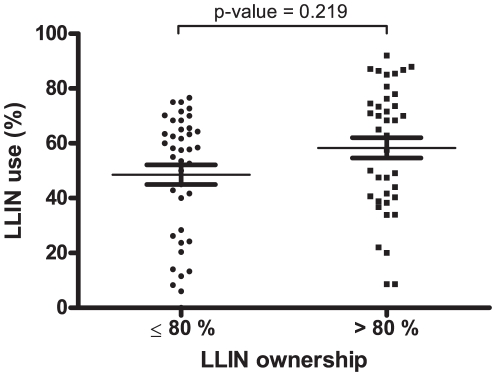
Mean village-survey LLIN use rates in children of under 5 years of age for ≤ and >80% LLIN ownership. LLIN: Long Lasting Insecticidal Net. P-value given according to binomial mixed-effect models analysing the probability of LLIN use >60% and taking into account repeated measurements.

A multivariate BME model was then developed to assess the combined effects of the explanatory variables on the probability of a LLIN use rate of over 60%. For the sake of consistency, only the surveys for which LLIN ownership was >60% (n = 79 over 84) were input (a non-null probability of LLIN use >60% was then always possible). The BME showed that the higher the level of biting nuisance (>1,456 bites per human per 100 nights, p = 0.056), the higher the probability of LLIN use >60% (Table 2). On the other hand, for the highest nocturnal temperatures (>22°C), this probability decreased significantly (p = 0.045). No relationship emerged between high LLIN ownership and the probability of LLIN use >60% (p = 0.236) confirming univariate analysis (Figure 5, p = 0.219).

**Table 2:** Binomial mixed-effect model to study the probability of achieving a LLIN use rate of over 60% in children of under 5 years of age, according to biting nuisance, nocturnal temperature and LLIN ownership (N = 79; number of groups = 27).

	Univariate analysis				Multivariate analysis			
Effects	Odds-ratio	95% Confidence interval		P-value	Odds-ratio	95% Confidence interval		P-value
Biting nuisance (bites of mosquitoes per human per 100 nights) ‡ $								
0–56 (21)	1				1			
57–447 (18)	1.0	0.26	3.80	1.000	0.78	0.18	3.46	0.747
448–1,456 (19)	1.8	0.50	6.46	0.368	1.76	0.43	7.19	0.428
1,457–6,419 (21)	**5.0**	1.35	18.55	**0.016**	**4.08**	0.97	17.23	**0.056**
Nocturnal temperature (°C) $								
14.5–19.5 (18)	1				1			
19.6–20.5 (19)	1.37	0.37	5.12	0.638	2.26	0.51	10.09	0.286
20.6–22 (22)	0.8	0.23	2.79	0.737	0.65	0.15	2.78	0.558
22.1–23.7 (20)	**0.2**	0.05	0.84	**0.028**	**0.20**	0.04	0.97	**0.045**
LLIN Ownership § $								
>60% and ≤80% (39)	1				1			
>80% (40)	1.75	0.72	4.27	0.219	2.06	0.62	6.85	0.236

‡ Biting nuisance is the human biting rate of all culicidae.

§ LLIN: Long Lasting Insecticidal Net.

$ the size of each category is indicated in parentheses.

## Discussion

### Malaria in Southern Benin during the dry season

The first part of this work aimed at investigating determinants of malaria disease in children of under 5 years of age during the dry season, after the nationwide distribution of LLINs in Benin. Malaria incidence varied between different villages in the OKT district from 0 to 23 malaria cases per 100 child-months: marked differences were even observed between neighbouring villages under three kilometres apart. This finding corroborates those of Djènontin et al. [7] who described marked spatial heterogeneity in the distribution and density of malaria vectors in this area.

These results show that the EIR of *An. funestus* correlate with the number of malaria attacks. This confirms previous works in Africa suggesting that *An. funestus* may play an important role in malaria transmission during the dry-hot season [20], [21], [22], [23]. In the OKT district, the highest malaria incidences were found in villages close to the types of permanent freshwater deposit (lakes and marshes in southeast and east) with vegetation, that provide ideal breeding sites for this species. In contrast, the component of transmission due to *An. gambiae s.s.* did not correlate with malaria incidence whatever the analysis method used (univariate or multivariate). It is important to note that the HBR for this species was low and its persistence through the dry season was dependent on occasional rain showers and the existence of permanent, domestic breeding sites [6], [24]. Because of the low number of *An. gambiae* caught, the precision of the values of EIR were low and therefore, the absence of correlation does not allow to rule out the role of *An. gambiae s.s.* in malaria incidence during the dry season.

### LLIN ownership and use

According to the WHO, a LLIN ownership rate of at least 80% is the minimum for good community protection against malaria [1]. In this study, we were not able to show any influence of high LLIN ownership on malaria incidence even when other thresholds were applied (N. Moiroux, unpublished data). This could be explained by the fact that LLIN ownership was not a good predictor of LLIN use. Conversely, our model showed that substantial LLIN use (>60%) had a significant impact on malaria incidence in the targeted population hence confirming previous observations [25]. Therefore, these results indicate that LLIN use should be preferred to ownership as indicator of malaria risk. Despite the hundreds of millions nets distributed in Africa since 2000, the estimated proportion of children of under 5 years of age sleeping under an effective net in sub-Saharan Africa is still low (35%) [1]. It will be a priority to improve this rate if the Millennium Development Goals for malaria control are to be achieved by 2015 [26]. Consciousness-raising campaigns should be maintained during the dry-hot season to promote LLIN use which remains the main means of community-based prevention in Africa.

This study also showed that mosquito-biting nuisance positively correlated with widespread LLIN use (>60%). This in agreement with Thomson et al. [27] who showed a strong association between net use and mosquito nuisance in Gambia where, as well as in other African countries [28], [29], anopheline species accounted for a large part of overall mosquito-biting nuisance and net use increased with the risk of being bitten by a malaria vector. In Southern Benin, malaria vectors are not the main mosquito nuisance and this explains why HBR for vectors does not correlate with LLIN use.

Furthermore, the highest nocturnal temperatures were found to be associated with low rates of LLIN use. This finding confirms results from interviews with populations in Burkina-Faso and Ghana [12], [13] who said they had trouble sleeping under a net on hot nights.

This study gives the first indications of the associations between the intensity of nuisance, nocturnal temperature and LLIN use, and confirms certain assumptions about seasonal variations in net use in Africa [6], [11], [12], [13], [14], [15].

### Implications for vector control and malaria modelling

In the OKT district (Southern Benin), *An. funestus* becomes the primary vector of malaria during the dry-hot season replacing *An. gambiae s.s.* Unfortunately, little is known about this species' biting behaviour, ecology and insecticide resistance status [10]. Some recent surveys in West Africa suggested that *An. funestus* may be resistant to pyrethroids, DDT and carbamates in Ghana [30], [31] but fully susceptible to pyrethroids in Burkina-Faso [32] and Benin (Corbel, unpub. data). Clearly more work is needed to characterize insecticide resistance mechanisms in *An. funestus* in West African countries.

According to the literature, when it is present, *An. funestus* can account for a significant component of malaria transmission [20], [21], [23], [33] and incidence in Africa. For efficient malaria control, it is therefore important to identify areas where this vector is present and abundant. Questions on its nocturnal biting habits should also be addressed, e.g. a study in Ghana [34] reported that its aggressiveness peaks in the last hours of mosquito collection (between 4 and 6 a.m.) which is consistent with our data (N. Moiroux, unpub. data). It is therefore likely that *An. funestus* continues to bite at dawn when people are no longer sleeping under mosquito nets. This could be an issue since current vector control strategies, relying on the use of residual insecticides (LLIN, IRS) target nocturnal, endophilic malaria vectors [12]. More knowledge on biting preferences, abundance, spatial distribution and resistance is needed so that *An. funestus* can be included in future models of malaria transmission. These models will have to integrate LLIN use but perhaps this could be replaced with proxy markers such as minimum temperature (which is easily ascertained) and nuisance. However, temperature should be used with care in transmission or disease models because of this parameter has disparate, poorly understood—and sometimes antagonistic—effects on LLIN use, vectors densities and transmission [35], [36], [37].

As recommended by the Malaria Eradication (MalERA) Consultative Group on Modelling, there is a need to understand better the effects of seasonality on malaria morbidity [38]. This study highlights the need to take into account the specificity of malaria epidemiology during the dry-hot season and get a better understanding of the factors that influence malaria incidence and net use. This will be important when it comes to developing more effective malaria modelling and mapping programmes. These findings should help National Malaria Control Programmes to implement more effective and sustainable malaria control strategies in Africa.
